# Recent advances and future challenges of tumor vaccination therapy for recurrent glioblastoma

**DOI:** 10.1186/s12964-023-01098-0

**Published:** 2023-04-12

**Authors:** Binghao Zhao, Jiaming Wu, Huanzhang Li, Yuekun Wang, Yaning Wang, Hao Xing, Yu Wang, Wenbin Ma

**Affiliations:** 1 Departments of Neurosurgery, Peking Union Medical College Hospital, Chinese Academy of Medical Sciences and Peking Union Medical College, Beijing, 100730 People’s Republic of China; 2 State Key Laboratory of Complex Severe and Rare Diseases, Peking Union Medical College Hospital, Chinese Academy of Medical Science and Peking Union Medical College, Beijing, People’s Republic of China

**Keywords:** Tumor vaccine, Immunotherapy, Recurrent glioblastoma, Clinical trial

## Abstract

Glioblastoma (GBM) is the most malignant CNS tumor with a highest incidence rate, and most patients would undergo a recurrence. Recurrent GBM (rGBM) shows an increasing resistance to chemotherapy and radiotherapy, leading to a significantly poorer prognosis and the urgent need for novel treatments. Immunotherapy, a rapidly developing anti-tumor therapy in recent years, has shown its potential value in rGBM. Recent studies on PD-1 immunotherapy and CAR-T therapy have shown some efficacy, but the outcome was not as expected. Tumor vaccination is the oldest approach of immunotherapies, which has returned to the research focus because of the failure of other strategies and subversive understanding of CNS. The isolation effect of blood brain barrier and the immunosuppressive cell infiltration could lead to resistance existing in all phases of the anti-tumor immune response, where novel tumor vaccines have been designed to overcome these problems through new tumor antigenic targets and regulatory of the systematic immune response. In this review, the immunological characteristics of CNS and GBM would be discussed and summarized, as well as the mechanism of each novel tumor vaccine for rGBM. And through the review of completed early-phase studies and ongoing large-scale phase III clinical trials, evaluation could be conducted for potential immune response, biosecurity and initial clinical outcome, which further draw a panorama of this vital research field and provide some deep thoughts for the prospective tendency of vaccination strategy.

Video Abstract

**Supplementary Information:**

The online version contains supplementary material available at 10.1186/s12964-023-01098-0.

## Importance of this study

1. We reviewed the literature concerning immune microenvironment of GBM, from the biological features of the immunosuppressive CNS to the mechanism of immunotherapy resistance in detailed stages, and focused on the newly emerging evidence.
2. We summarized the differences between newly diagnosed GBM and rGBM through immunological features, highlighting the uniqueness of rGBM and discussed the possible connection between the unique immunological characteristic and the failure of present clinical trials on immunotherapy of this tumor.
3. We comprehensively reviewed the mechanism of each novel tumor vaccine for rGBM, and explained the reason we believed in these vaccines despite the present challenges.
4. We conducted a detailed summary on the completed and ongoing RCTs of tumor vaccines for rGBM, from preclinical trials to large-scale phase III trials, analyzing the clinical outcomes, and pointing out the achievements as well as problems for all vaccines.
5. We depicted a tumor vaccination landscape for rGBM treatment and provided some deep thoughts for the prospective tendency of vaccination strategy, from basic research to clinical application.

## Introduction

Glioma is the most common primary tumor in central nervous system (CNS), with an incidence rate of about 8/100000 people worldwide [1, 2]. There are more than ten subtypes of glioma according to the 2021 WHO Classification of Tumors of the Central Nervous System [3, 4], and among which, glioblastoma (GBM) has occupied a crucial role because of its highest malignancy and a 60% proportion of all patients [5]. The standard therapy, established by Stupp in EORTC-26981 trial in 2005, consists of grass total surgical resection, concurrent radiotherapy combined with temozolomide (TMZ) and adjuvant TMZ [6], and with the encouraging result of a 20.9-month overall survival (OS) in EF-14 trial [7], the latest standard of care has added concurrent treatment with alternating electric fields and adjuvant TMZ to the first-level choice as well as the EORTC-NCIC study-based adjuvant involved-field RT with concurrent and adjuvant TMZ [8]. However, the median OS is still about 14.4–16.7 months in most randomized clinical trials (RCTs) [2, 9, 10] with an almost inevitable tumor recurrence [11]. And for patients with recurrent GBM (rGBM), the resistance to radiotherapy and chemotherapy could occur on a much larger scale, with only fewer than 30% of the patients qualified for a second surgery [12, 13]. Bevacizumab (BEV) is a widely approved treatment for improving progression-free survival (PFS), but it failed to extended OS in the studies [14]. As a result, the median OS of these patients ranges from 4.7 to 11.4 months based on highly individualized therapeutic choices in different RCTs [15, 16]. Therefore, GBM is still one of the most dangerous cancers with leading mortality, especially for rGBM, which has no established standard care till now, making it an urgent need for researchers to explore novel therapeutic targets and plans [17].

Immunotherapy has been a rising star in the field of tumor treatment in recent years [18], which is defined as a biological treatment using substances to stimulate or suppress the immune system, helping human body against cancer, infection, or other types of diseases according to National Cancer Institute (NCI) [19]. For cancer treatment, immunotherapy aims to generate a tumor-specific immune response to selectively eliminate tumor cells [20], which can be divided into two parts: active immunotherapy and passive immunotherapy [21, 22]. The active immunotherapy induces certain immune responses against tumors by injection of exogenous antigens, such as vaccines including peptide vaccines and cell-based vaccines, while passive immunotherapy kills tumor cells by injecting exogenous immune substances without a direct activation of the body’s immune system, which includes but not limited to antibody therapy and adoptive immunotherapy [23].

Based on the different immune molecular pathways, target proteins and mechanisms, there have been several kinds of treatments that have been applied to clinical practice or achieved the clinical trial for further development, including tumor vaccines, adoptive immunotherapy, immune checkpoint inhibitor, virus therapy and intratumoral injection etc. [24–27]. As the first-developed immunotherapy, tumor vaccination has been one of the most important approaches for researchers to regulate human immune system to strengthen the local immune response and consequently, reach a therapeutic reaction [28, 29]. Novel treatments have achieved much impressive therapeutic outcome in some specific tumors in recent years. Immune checkpoints PD-1/PD-L1 was found promising through clinical trials in non-small cell lung cancer, [30–33],while chimeric antigen receptor T-cell (CAR-T) therapy has been a greater hotspot for the investment in immunotherapy because of its remarkable efficacy in hematological malignancies However, the profound therapeutic effects were limited in solid tumor till now. In recent researches on CAR-T therapy, not only the outcome of survival didn’t meet the expectation in CNS tumor-related studies [34], the treatment also showed severe neurotoxicity through the activation of the microglia and astrocytes and some life-threatening graft-versus-host responses [15, 35–38]. The disappointing progress could be attributed to the unique immunosuppressive microenvironment of brain tumors and CNS, which on the other hand, pointed out a greater possibility of success in tumor vaccination [17, 18]. In this review, we introduced the immunological characteristics of CNS and GBM, especially the uniqueness of rGBM, and the basic mechanism of tumor vaccination to explain the reason we believe in this traditional way of immunotherapy and the present and potential challenges. Then we would review the completed and ongoing clinical trials on tumor vaccination for rGBM in recent years, hoping to make a panorama of this vital research field and provide some deep thoughts for the future progress.

### Immune microenvironment of GBM

**Fig. 1:**
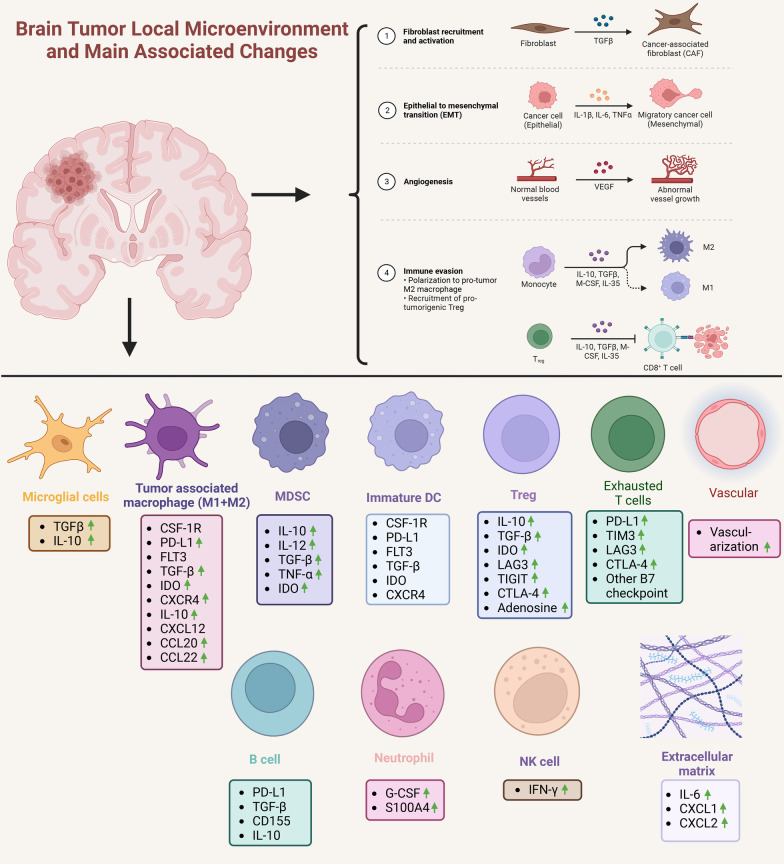
Glioma local microenvironment and main associated changes. The glioblastoma (GBM) has highly immunosuppressive tumor microenvironment (TME) consisting of considerable cells, cytokines, chemokines and microvessels. TGF-β will transfer the fibroblast into cancer-associated fibroblast (CAF); there will be more epithelial mesenchymal transition (EMT) under IL-1β, IL-6 and TNF-α; with VEGF, there will be more abnormal vessel growth. With IL-10, TGF-β, M-CSF and IL-35, M2-macrophage polarization will be enhanced and regulatory T cells (Tregs) will inhibit immune activity of CD8 + T cells by secreting IL-10, TGF-β, M-CSF and IL-35. The tumor cells highly express immune suppressive factors like programmed cell death ligand 1 (PD-L1), IDO and decreases the level of MHC to inhibit tumor antigen recognition and presentation. In GBM TME, the microglial cells always downregulate potential immune response and promotes systematic immunosuppression by secreting TGF-β and IL-10. Tumor-associated macrophages (TAMs) has two subtypes, namely immunopromoting subtype (M1) and immunosuppressive subtype (M2). TAMs mediate and balance tumor immune activity by highly expressing PD-L1 and secreting TGF-β, IDO, CXCR4, IL-10, CXCL12, CCL20, CCL22 etc. MDSCs highly secrete IL-10, IL-12, TGF-β, TNF-α, IDO to inhibit immunotherapy response. Immature DCs can secrete some factors and express PD-L1, however, role of immature dendritic cells (DCs) is not determined. Tregs mediate immunosuppressive effects through upregulation of various soluble factors, immune checkpoints and metabolic pathways. Due to the increased levels of checkpoint exhaustion molecules, exhausted T cells downregulates immune response. Neutrophil and natural killer cells (NK cells) participates in the regulation of immunotherapy by upregulating G-CSF, S100A4 and IFN-γ, while clear role of B cell in GBM TME is not well established. Extracellular matrix (ECM) also serves as an important component in GBM TME. Vascularization is observed to be reinforced in GBM immunosuppressive TME, therefore anti-vascularization can be useful target to treat GBM. Immune cells, for example DCs, can migrate via tumor draining lymph nodes of the brain to deep cervical lymph nodes and promote tumor antigen to promote an adaptive antitumor immune response. The process can also be suppressed by the local immunosuppressed TME. On one hand, the bone marrow can restore and release suppressed T cells, on the other hand, chemotherapy (eg., TMZ) to GBM can induce lymphopenia that is exacerbated by bone marrow sequestration of T cells. Specific T cells to tumor antigens can be destroyed by spleen. Green arrow indicates the factors or activities are upregulated. CSF, colony stimulating factor; APC, antigen-presenting cells; IDO, indolamine 2,3-dioxygenase; MHC, major histocompatibility complex

The development of immunotherapy in glioma have experienced a long and winding road in the past decade [39]. As the most aggressive and malignant form of primary CNS tumor, GBM has unique molecular features and biological properties, which has led to its tendency of recurrence, strong infiltration among brain tissues and high resistance to chemotherapy, radiotherapy and immunotherapy [40]. The unique immune microenvironment of GBM has been found to play a crucial role. Generally speaking, GBM has been considered as a “cold” tumor immunologically, with multiple mechanism against human immune system [41, 42] (Fig. 1). Such extensive immunosuppressive mechanisms then enable GBM to be segregated by the local and systematic response based on its location in the CNS [43]. However, through numerous studies, more evidence confirmed that GBM could be recognized by human immune system and not totally unaffected by the attack of the immune system, showing some immunotherapy-responsive tumors display initially [43, 44]. The crux is its strong ability to keep away from immunosurveillance, escaping followed-up immunological pressure, while its immune microenvironment highly contributes to this process [45]. To be detailed, several pathways are involved in the immunotherapy resistance of GBM, which cover the mechanisms found in other solid tumors, as well as some unique strategies consequence of its location in the CNS [46].

Systematic immunosuppression is a major feature of GBM and its microenvironment, which causes immunological dysfunction in a wide range of patients [47]. The microenvironment of GBM is generally infiltrated with immunosuppressed immune cells. A study has shown that a relatively high ratio of CD4 + tumor-infiltrating lymphocytes to CD8 + tumor-infiltrating lymphocytes was observed around GBM, which was proved to be a signal of poorer overall survival [44, 48]. Regulatory T cells, another subtype of immune cells which would turn the immune microenvironment less responsive to immunotherapy, were found highly infiltrated in GBMs rather than in low grade gliomas, and expressed the transcription factor FoxP3 [44]. Meanwhile, GBM could secrete paracrine immunosuppressive mediators, such as l-Tryptophan (Trp) and indoleamine-2,3-dioxygenase 1 (IDO1) [49]. Sphingosine-1-phosphate receptor 1 (S1P1), another functional protein on the T cell surface, could direct to the sequestration of T cells in bone marrow, leading to systemic immunosuppression [43].

The intrinsic resistance is another important mechanism for GBM. In a study, researcher took samples from spatially isolated regions in 11 different GBMs, several molecular subtypes were found present within one same tumor, showing great molecular heterogeneity [50, 51]. Such heterogeneity could lead to the selective destruction of treatment-susceptible clones, and finally prevent the initiation of an immune response. And for the process of recurrence, the driver clonal mutation would be a subclone from the primary one instead of the initial clonal mutation of the primary GBM [51], resulting in the failure of some targeted therapies for rGBM based on the tumor genome at primary diagnosis [52, 53]. For example, clinical trials of EGFR and EGFR vIII could be effective for the initial tumor but not responsive in the recurrent one due to its immune evasive trait [54]. Other immune pathways are also affected through different mechanisms in GBM, such as adaptive resistance deactivating tumor-infiltrating immune cells [55, 56], developing resistance protecting the tumor from being eliminated in the face of attack by the immune system [57, 58]. Generally, GBM cells can express various regulators to modulate their immune microenvironment, and therefore avoid complement attack, gain adaptive resistance and enhance immunosuppression.

### The immunosuppressive CNS

As the living environment of GBM, CNS is tightly connected to the tumor and its progression. On the one hand, CNS brings GBM some unique molecular and histologic features through the specific types of cells that only exist in CNS and their own function [59, 60]. On the other hand, GBM shares a similar microenvironment with the whole CNS, which turns the special immunological feature of CNS for preventing itself to a great obstacle for immunotherapy to come into effect [61, 62]. The CNS has long been considered as an immune privileged site, with the unique biological structure made up of a well-developed blood–brain barrier (BBB) and surveillance system with microglia. And the BBB is a network mainly based on endothelial tight junctions with non-fenestrated cells, finally presented as tissue and blood vessels [60]. These semipermeable connections made it possible to prevent leakage of hydrophilic solutes as well as allowing the exchange of hydrophobic ones and active transport of circulating nutrients [63]. However, with the deeper exploration of CNS, researchers found that BBB could have a better permeability when the immune system aroused a full-scale systemic response to antigens under some pathological states such as an infection in CNS [64, 65], as well as other events that were able to induce CNS inflammation including autoimmunity, abnormal metabolites, brain trauma or stacking of misfolded proteins [66, 67]. The discovery has provided a theoretical possibility of the immunotherapy in CNS, that through peripheral immune cells carrying the essential substrate for immunotherapy and crossing the BBB as soon as endogenous potential harmful particles are detected, the effective cytokines and other immune-mediated small particles would direct toward the focus in brain, such as brain tumors [18, 64].

### The unique immunological feature of rGBM: a comparison of GBM and rGBM

There hasn’t been much research on the features of rGBM, since the greater heterogeneity it showed compared to newly diagnosed GBM and the difficulty of rGBM sample collecting [68]. However, some features were found common in a few researches. A study showed that rGBM has nearly double IDH-1 mutation rate than newly diagnosed GBM [69]. Another study focused on the biological characteristics of rGBM. By using the four subtypes of GBM for classification, research found a difference that the proportion of the classical subtype in rGBM was lower than that in primary GBM [70]. Other researches illustrated that rGBM were likely to be pro-neural (PN) subtype, in which of monocyte, regulatory T cell markers and immune checkpoint receptors were decreased, attenuating the immunosuppressive influence of rGBM on cytotoxic T cells [44, 71, 72]. TP53 mutation occurred much in rGBM, which is known to affect the expression of the immune checkpoint receptors cytotoxic T lymphocyte-associated antigen-4 (CTLA-4) and programmed death-ligand 1 (PD-L1) [70, 73, 74].

The uniqueness of rGBM could be shown in another critical aspect as the immune microenvironment. A single-cell analysis of primary and recurrent GBM samples showed that they share similar immune signatures in general, but a remarkable difference appeared on glioma-associated microglia/macrophages (GAM), the proportion of which decreased more than 50 percent in rGBM samples. And some undefined CD45 + immune cells took up a much greater part among the immune cells of rGBM, which remained further classification. Regulatory T cells (Tregs) could be an important obstacle of anti-tumor treatment because of its mediation of immunosuppressive effects through upregulation of various soluble factors, immune checkpoints and metabolic pathways. The proportion of Tregs was higher than normal in tumor samples of all patients, while there was not a significant difference between the primary and recurrent ones [75, 76]. Meanwhile, the spatial organization might be another change occurred in the tumor immune environment for recurrence, especially for T cells. In a study conducted with high-dimensional cytometry, T cells were found enriched and activated in perivascular regions, where there were fewer regulatory T cells and more activated macrophages [77]. Another comparative study on tumor immunologic features found that in comparison with the tissue sample from newly diagnosed GBM, tumor tissue of rGBM showed much higher levels of infiltration of CD4 + T cells, CD8 + T cells, CD68 + macrophages, and CD163 + macrophages [78], however no significant difference was found in the CD8 to CD4 ratio of those two groups [79]. And the immune cells infiltrated in recurrent tumors accumulated in the perivascular region with a majority of CD4 + T cells. The increase of immune cell infiltration in rGBM seems to be unmatched with the poor survival [80, 81], which might be explained as the large percentage of the increasing accumulating immune cells could be transferred to the exhausted subset or converted to immunosuppressive ones, so that more brain edema and other severe events came up instead of a better microenvironment for immune response.

### Tumor vaccine in GBM

**Fig. 2:**
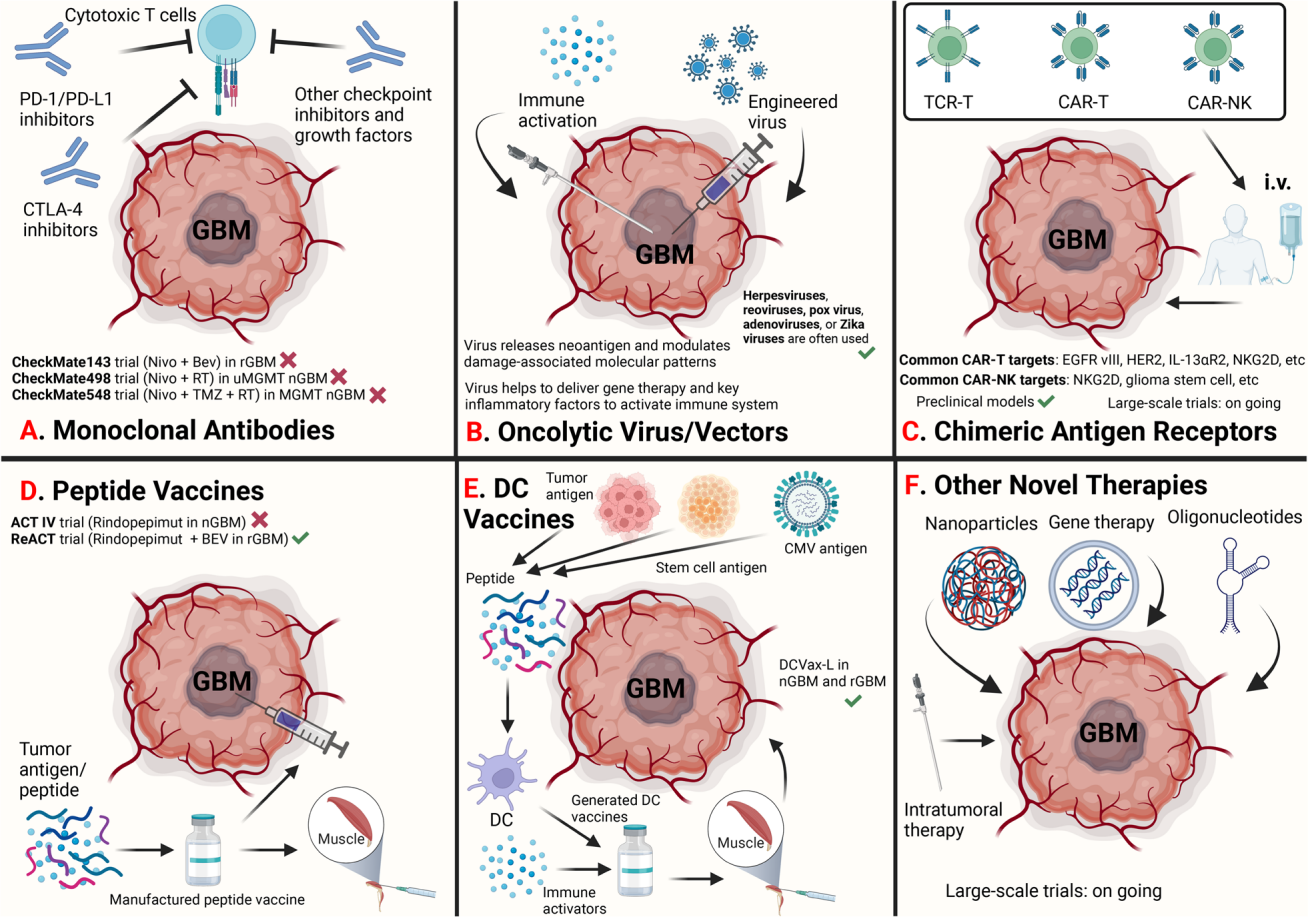
Major available immunotherapies for newly diagnosed and recurrent glioblastoma. **A **Treatment of monoclonal antibodies. There are three phase III clinical trials involving immune checkpoint inhibitors on GBM, namely CheckMate143 in rGBM, CheckMatre498 in uMGMT nGBM and CheckMate548 in MGMT nGBM. However, all the three clinical trials failed to prolong OS of nGBM/rGBM. **B** Treatment of oncolytic virus/vectors. Virus potentially releases neoantigen and modulates damage-associated molecular patterns, it also helps to deliver gene therapy and release key inflammatory factors to activate immune system. Herpesviruses, reoviruses, pox virus, adenoviruses and Zika viruses are commonly used in vaccines in clinical manner. Briefly, virus vaccines and vectors have showed favorable anti-tumor activity in preclinical models and small clinical trials. **C** Treatment of chimeric antigen receptor. Chimeric antigen receptor (CAR) therapies mainly include CAR-T, TCR-T and CAR-NK. Common CAR-T targets involve EGFR vIII, HER2, IL-13αR2, NKG2D etc., common CAR-NK targtes involve NKG2D, glioma stem cell etc. CAR therapies demonstrate promising efficacy in preclinical glioma models, the large-scale clinical trials are still ongoing. **D** Treatment of peptide vaccines. EGFR vIII is also regarded as a target for peptide vaccine in glioma, the ACT IV trial administrates Rindopepimut in nGBM, the ReACT trial uses Rindopepimut to treat rGBM. OS of rGBM is prolonged in ReACT trial. **E** Treatment of DC vaccines. Tumor antigen, stem cell antigen and CMV antigen can be degraded to peptide, distinct peptide will invoke DCs to secrete immune activators to enhance the anti-tumor immunity. After the process by the peptide, sensitive DCs will be selected to generate DC vaccines. A phase III randomized controlled trial conducted on nGBM and rGBM reveales DCVax-L prolongs the OS with acceptable toxicity. **F** Other novel therapies include nanoparticles therapy, gene therapy and oligonucleotide therapy. The check mark in green indicates OS of glioma patients can be prolonged in clinical trials; the cross in red indicates OS of glioma patients can not be significantly prolonged. PD-1, programmed cell death 1; PD-L1 programmed cell death ligand 1; GBM, glioblastoma; nGBM, newly diagnosed GBM; rGBM, recurrent GBM; uMGMT, MGMT promoter unmethylated; MGMT, MGMT promoter methylated; DCs, dendritic cells; CAR, chimeric antigen receptor; TCR, T cell receptor-T; NK cells, natural killer cells; CMV, cytomegalovirus; OS, overall survival

There are many types of immunotherapies for glioma/GBM, of which tumor vaccine can target tumor antigens and amplify anti-tumor immune response to achieve therapeutic effect [29, 82] (Fig. 2). The effective components of a tumor vaccine are mainly two parts, tumor antigen, and immune adjuvant. Tumor antigen is the cornerstone of a tumor vaccine, therefore, choosing the appropriate tumor antigen is the first step for the establishment of anti-tumor immune response [83]. And tumor-associated antigens (TAAs) have occupied a central place in the process. A number of studies have examined the expression of TAAs in GBM, which has shown the possibility of several potential candidates for vaccine-directed immunotherapy [84, 85]. As a result, dozens of GBM-related TAAs have been found till now, including ACTL8, CTCFL, Opa interacting protein 5 (OIP5), XAGE3, CD133, epidermal growth factor receptor vIII (EGFR vIII), Interleukin-4 (IL-4), gp100, survivin, Interleukin-13 receptor subunit alpha-2 (IL-13Rα2), Human Epidermal Growth Factor Receptor 2 (HER2), Human Chitinase-3-like Protein 1 (YKL-40), and erythropoietin-producing hepatocellular receptor tyrosine kinase class A2 (EphA2) [29] with evaluation in early-stage therapeutic preclinical trials demonstrating the safety and immunogenicity in human body. However, confirmation is still necessary to prove the existence of a therapeutic window, where the vaccines against the tumor could induce sufficient immunity to achieve clinical efficacy without severe systemic autoimmune manifestations while maintaining a low level of expression in normal tissues. Viral antigen, which has the longest history for an application that could trace back to 1891, is a special class of TAAs which are exogenous structures to the host immune system [86]. The inherent immunogenicity makes it an excellent target for tumor-directed immunotherapy. Human cytomegalovirus (CMV) antigens were used most widely in the present studies [85, 87]. The other important subgroup of tumor antigen is the tumor-specific antigen. It precisely expressed within tumor cells while a shared expression could hardly be found in normal tissues, such as EGFR vIII [37].

Immune adjuvant can do help to the activation of dendritic cells to assist in enhancing antigen presentation and costimulatory signals, which are generally pattern recognition receptor agonists on the surface of antigen presenting cells (APCs) [88]. There are five ways for tumor antigens and immune adjuvants to coactivate the tumor-related immune response, including proteins/peptides, tumor cells, APCs, viruses and nucleic acids, each of which has its applicable situation [20].

### Clinical relevance

The clinical application of tumor vaccine in brain tumors could trace back to the 1980s. In a case report, Bergquis et al. [89] injected the cell wall of Bacillus Calmette Guerin (BCG) vaccine into patients with glioma. The therapy was confirmed to be effective for patients’ overall survival in another larger pilot study in 1983 [90]. Some researchers tried to combine a *Serratia marcescens*-derived vaccine with radiothera py to activate the patient’s immune system, which showed certain immune responses and tolerance in the experienced patients [91]. A phase I/II trial of intravenous NDV-HUJ oncolytic virus was conducted among patients with rGBM, in which the virus was expected to infect human cells as well as tumor cells to prevent the growth of tumor [92]. These vaccines in the early time were all non-specific, hoping to achieve the therapeutic effect through stimulating the whole immune system so as to activate its potential to kill tumor cells. After decades of development, there have been more than 50 ongoing registered randomized clinical trials for tumor vaccination therapy in GBM. The vaccines could be divided into four main subtypes, known as peptide vaccines, cell vaccines, DNA vaccines, and mRNA vaccines [42]. Peptide or DNA vaccines have the TAAs or DNA injected to elicit adaptative immune response. Cell vaccines, mostly DC vaccines, on the other hand, based on the cells stemmed from peripheral blood mononuclear cells (PBMCs), which can be prepared with tumor antigens. mRNA vaccines encode their own tumor antigens through mRNA, and the antigens sequentially fill the viral vectors for induction of immune responses [93]. However, most of the studies have not met the expectation, from the safety and toxicity of vaccines to benefit in survival and improvement of patient’s quality of life. Moreover, a major proportion of the clinical trials have been conducted on patients with newly diagnosed GBM, which has a larger patient cardinality. For rGBM researches, the recruitment of patients could be difficult, and lots of patients have already been under a poor physical condition, making it too dangerous to enroll in the trials. As a result, most completed or ongoing clinical trials stayed in phase I, especially for those completely aiming at patients with rGBM (Table 1) [94]. Only 4 vaccination agents, Rindopepimut, DCvax, HSP96 and PPV have reached the stage of phase III clinical trial [95–97].

**Table 1:** Available completed and ongoing clinical trials of tumor vaccine therapy for recurrent glioblastoma

Vaccine and treatment	Vaccine type	Trial id	Phase of clinical trial	Tumor types	Enrollment	Study design	Main conclusion	Reference
Rindopepimut + TMZ	EGFR-VIII peptide vaccine	NCT01498328 (ReACT trial)	II	Relapsed EGFR vIII-positive GBM	73	Open label Double-blind Two arms	Primary outcome, PFS6 was 28% (10/36) for rindopepimut compared with 16% (6/37) for control (P = 0.12, one-sided). Secondary endpoints favored the rindopepimut group, including a statistically significant survival extension, a higher overall response rate of 30%, a longer median duration of response, and the better ability to discontinue steroids for ≥ 6 months	[103]
HSPPC-96	HSP vaccine	NCT02722512	I	Recurrent resectable intracranial GBM	12	Open label Dose escalation Cohort expansion	No adverse events attributable to the vaccine were found. Testing of peripheral blood leukocytes before and after vaccination revealed a significant peripheral immune response specific for the peptides bound to HSP-96, in 11 of the 12 patients treated. The response time lasted 47 weeks for those patients having positive immune response	[105]
HSPPC-96	HSP vaccine	NCT00293423	I/II	Relapsed GBM	41	Open label Dose escalation Single arm	Median overall survival was 42.6 weeks (95% CI 34.7–50.5). Twenty-seven (66%) patients were lymphopenic prior to therapy, and patients with lymphocyte counts below the cohort median demonstrated decreased overall survival. No treatment-related severe side effects	[104]
HSPPC-96 + Bevacizumab	HSP vaccine	NCT01814813	II/III	Surgically resectable rGBM	90	Open label Double-blind Three arms	Ongoing	[109]
SruVaxM + Sargramostim	survivin vaccine	NCT01250470	I	Survivin-positive recurrent glioma	9	Open label Dose escalation	SurVaxM was well tolerated with mostly grade one adverse events and no serious adverse events attributable to the study drug. 6 of 9 had a cellular response and 3 of 9 achieved a local response. The median PFS of all patients was 17.6 weeks, and the median OS reached 88.6 weeks with 7 patients surviving more than 12 months	[111]
SurVaxM + Pembrolizumab	Survivin vaccine	NCT04013672	II	rGBM	40	Open label Double-blind Two arms	Ongoing	[112]
SL-701 + poly-ICLC	Glioma-associated antigen vaccine	NCT02078648	I/II	rGBM	74	Open label Dose escalation Single arm	Ongoing	[115]
IMA950/Poly-ICLC + Pembrolizumab	Multipeptide vaccine	NCT03665545	I/II	Relapsing GBM	24	Open label Dose escalation Two arms	Ongoing	[116]
EO2041 + Nivolumab + Bevacizumab	Multipeptide vaccine	NCT04116658	Ib/IIa	Progressive or first recurrent GBM	76	Open label Dose escalation Three arms	All patients have been well tolerated through injections. The biosecurity feature of EO2401 was almost the same as nivolumab. Robust immune response has been observed in EO2401 plus nivolumab group. The median PFS of EO2401 combined with nivolumab and standardized bevacizumab group was 5.5 months, and median OS was 12.2 months	[117]
HLA-A24–restricted vaccine candidates (ITK-1)	Multipeptide vaccine	UMIN000001243	I	Recurrent or progressive supratentorial GBM	12	Open label Dose escalation Cohort expansion	No serious adverse drug reactions were encountered, and treatment was well tolerated. The vaccine induced dose-dependent immune boosting with a response rate of 16.7%. The recommended dose of ITK-1 peptides is 3 mg/peptide. The median PFS is 2.3 months	[118]
DSP-7888	Peptide vaccine	NCT02498665	I	Recurrent or advanced AML, MDS, GBM, melanoma, NSCLC, ovarian cancer, pancreatic cancer, sarcoma, or renal cell carcinoma	24	Open label Dose escalation	DSP-7888 Dosing Emulsion was well tolerated, with no dose-limiting toxicities. Higher WT1-specific cytotoxic lymphocyte induction was noted with intradermal injection. 7 rGBM patients showed robust immune response	[120]
DSP-7888	Peptide vaccine	NCT03149003	III	Recurrent or progressive supratentorial GBM	236	Open label Double-blind Two arms	Ongoing	[121]
HLA-A*2402-restricted, modified 9-mer WT1 peptide vaccine	Multipeptide vaccine	–	II	rGBM	21	Open label Single arm	The overall response rate was 9.5% and the disease control rate was 57.1%, with 2 patients showing partial response, 10 patients under stable disease, and 9 patients under progressive disease. The median PFS was 20.0 weeks, and the 6-month (26-week) PFS rate was 33.3%	[122]
PPV	Multipeptide vaccine	–	III	HLA-A24–positive rGBM	88	Open label Double-blind Two arms	The median OS was 8.4 months in PPV group versus 8.0 months in placebo group and no significant difference on median PFS or 1-year survival rate between the two groups	[96, 145]
DCVax-L	DC vaccine	NCT00045968	III	Newly diagnosed GBM and rGBM	331	Open label Double-blind Two arms	A group of 64 patients receiving only SOC plus placebo until recurrence, then receiving DCVax-L was defined as placebo arm crossovers. Its median OS was OS in rGBM, which was the secondary endpoint. The median OS was 13.2 months while the external control group was 7.8 months. The 24- months overall survival was 20.7% versus 9.6% and the 30 months survival was 11.1% versus 5.1%	[95, 126]
αDC1 loaded with synthetic peptides + poly-ICLC	Peptide-pulsed DC vaccine	NCT00766753	I/II	Recurrent malignant gliomas	22	Open label Dose escalation Single arm	The protocol was well-tolerated. 9 of 22 patients reached a PFS of more than 12 months, with positive immune responses observed in 58% of the patients. One patient demonstrated a sustained complete response	[134]
Autologous DC vaccine pulsed with lysate derived from a GBM stem-like cell line + Bevacizumab	Peptide-pulsed DC vaccine	NCT02010606	I	Newly diagnosed GBM and rGBM	35	Open label Dose escalation Cohort expansion	25 patients diagnosed with rGBM were all well tolerated. No severe adverse events occurred. The median OS and PFS were 11.97 months and 3.23 months, and 6-month PFS was 24%, all better than the average	[135]
GSC-DCV	Peptide-pulsed DC vaccine	–	II	Newly diagnosed GBM and rGBM	21	Open label Dose escalation Two arms	10 patients with rGBM have a median OS of 10.7 months, and a median PFS of 6.9 month. Subgroup analysis was limited due to the study size	[136]
Gliadel Wafer + tumor lysate-pulsed DC vaccine	Tumor lysate-pulsed DC vaccine	–	I	Primary and recurrent malignant giloma	28	Open label Dose escalation Cohort expansion	The protocol was safe and elicited modest immunogenicity. 17 patients with rGBM reached a median OS of 10.9 months and a median PFS of 1.9 months	[138]
WT1-pulsed DC vaccine	Tumor lysate-pulsed DC vaccine	–	I	Recurrent malignant glioma	10	Open label Dose escalation	The WT1-pulsed DC vaccination therapy proved its safety, immunogenicity, and feasibility. All the enrolled patients finally had a progression in tumor	[139]
Adjuvant DC-based immunotherapy	Tumor lysate-pulsed adjuvant DC vaccine	–	II	Relapsed GBM	56	Open label Three arms	The median OS and PFS was about 9.6 months and 3 months, with a 2-year OS rate of 14.8%. To make a faster DC vaccination schedule with tumor lysate boosting was likely to improved PFS	[141]
ERC1671 + bevacizumab	Whole tumor vaccine	NCT01903330	II	rGBM	9	Open label Double-blind Two arms	The median OS of ERC1671 plus bevacizumab group was 12 months, compared to 7.5 months in the placebo plus bevacizumab group. The increasing maximal CD4 + T-lymphocyte leads to longer survival in ERC1671 group	[143]
ATL-DC	Tumor lysate-pulsed DC vaccine	NCT04201873	I	Surgically accessible rGBM	40	Open label Dose escalation Cohort expansion	Ongoing	[144]

### EGFR vIII peptide vaccine

EGFR vIII is a deletion mutation that generates a novel extracellular tumor-specific epitope. It is heterogeneously expressed in approximately one-third of the GBM population, and is not found in any normal tissues [98]. The mutation could enhance tumorigenicity and tumor cell migration by encoding a protein with an active tyrosine kinase. Preclinical studies also found it related to radiation and chemotherapeutic resistance to tumor cells [99]. And in a study on patients with GBM surviving more than 1 year, the expression of EGFR vIII was found to be an independent negative prognostic marker of survival, indicating it as a key potential target for anti-tumor immunotherapy [100]. Rindopepimut is a vaccine with 14 amino acid peptides from EGFR vIII encircling the mutation site and conjugated to keyhole limpet hemocyanin (KLH). In a phase II/III single-armed multicenter trial, the ACT III trial, patients with newly diagnosed GBM went through vaccination with rindopepimut combined with TMZ. 65 patients with EGFR vIII-positive GBM were recruited, with a median overall survival (OS) of 21.8 months and a 3-year survival rate of 26%, which significantly prolonged the median survival of patients, proving the great efficacy of this vaccine [101]. For rGBM, rindopepimut was treated as an addition to BEV, a VEGF receptor inhibitor that has been proved to extend the progression-free survival (PFS) of patients with GBM in a randomized phase II study. 70 patients were matched 1:1 randomly and received BEV plus rindopepimut or KLH, and BEV respectively. The objective response rate of the combined therapy group was 23%, 3 percent higher than the BEV group, indicating that rindopepimut had a positive anti-tumor effect in patients with rGBM. Despite the clinical trial ongoing with the follow-up of survival outcome, rindopepimut has shown a significant improvement of the overall survival in this context, with a median OS of 12.0 months in rindopepimut group (95% CI 9.7–NA) compared to that of the KLH group, which is 8.8 months (95% CI 6.8–11.4) [99, 102]. No patients possessed EGFR vIII-specific immune responses in advance of vaccination, proving the solid correlation between the induction of these responses and the extended OS.

A large-scale phase III study, the ACT IV trial has been completed subsequently, as a pivotal randomized placebo-controlled clinical trial in GBM. A sum of 745 patients were enrolled into rindopepimut plus TMZ or control (KLH) plus TMZ group, and their median OS were 20.1 months versus 20.0 months, showing no significantly difference [97]. Although rindopepimut didn’t extend survival in patients with newly diagnosed GBM as expected, researchers still looked forward to its potential efficacy within rGBM. In the newly released ReACT trial, 73 patients with rGBM were randomized into 36 rindopepimut group and 37 control group. The tolerability was first examined, as main toxic adverse events were transient, low-grade local reactions. As the primary endpoint, the 6-month PFS rate was 28% for rindopepimut, compared with 16% for control. Secondary and exploratory endpoints further demonstrated the advantages of the rindopepimut group, including a statistically significant survival extension, a higher overall response rate of 30%, a longer median duration of response, and the better ability to discontinue steroids for ≥ 6 months, which is 33–0% among all patients [103]. Despite more validation required due to the limited sample size and potential heterogeneity, it was still strong evidence to support the therapeutic effect of rindopepimut in patients with rGBM.

### HSP vaccine

Heat shock protein (HSP) vaccine was another immunization approach. Heat shock protein is a kind of protein that is widely found in microorganisms, plants and animals, which has molecular chaperone activity to inhibit the denaturing of biological macromolecules affected by the ambient temperature as well as oxygen content and ions [89, 104]. Therefore, HSPs could reassemble reducible misfolded proteins, and guide the degradation of unreducible ones, which is predicted to be upregulated in tumor tissues where more abnormal proteins would appear and translate. The core anti-tumor immunological function of HSP is to combine with nascent proteins to extensively activate innate and acquired immune systems in human body, and therefore, enhance tumor immunogenicity and regulate the immune response. However, only HSP gp 96, HSP 90, HSP 70, HSP 110, and HSP 170 have been found emerging such immunogenic response through researches. And among which, HSP96 is the subtype most closely related to glioma, which is related to EGFR-VIII, TERT, P53, CDK4, MAPK, PI3K and many other molecules and signaling pathways [105]. The HSP96 complex is first bound to CD91 on APCs, and brain tumor-derived HSP96 is internalized, which then leads to the presentation of HSP96-chaperoned tumor antigen on class I and class II major histocompatability complexes (MHC) and robust immunogenicity. The advantage of HSP vaccine, compared to other tumor vaccines, is its highly specificity of the interaction between HSPPC-96 and APCs, and therefore, better eliciting robust CD4 + and CD8 + T-cell immune responses. A phase I clinical trial with HSP96 involved 12 patients with rGBM who had undergone surgical treatment for dose escalation [105, 106]. The adverse event ranking first was mild injection site erythema and no serious adverse events occurred with this vaccine, while the response time lasted 47 weeks for those patients having positive immune response. The subsequent single-arm phase II trial involved 41 patients with relapsed GBM, showing that the median OS of the HSP96 group was 42.6 weeks after the application of vaccine without serious side effects, similar to the previous research [107, 108]. Additionally, patients with reduced lymphocyte count were found to have a poorer OS. The randomized phase III trial supported by the Alliance Consortium is already underway with a primary purpose of judging whether there is an OS advantage of HSPPC96 combined with BEV, given concomitantly or at the point of progression in patients with rGBM [109].

### Survivin vaccine

Survivin is an intracellular anti-apoptotic protein, which is overexpressed within brain tumors. It can inhibit caspase activation and as a result, regulate cell division. The immunogenicity of survivin could be proved by survivin-specific cytotoxic T lymphocytes and humoral immune response with anti-survivin antibodies detectable in serum, both being found in a certain of patients [110]. It wasn’t frequently detected in normal tissues of human, therefore, making it an ideal vaccination target. SurVaxM, a peptide vaccine targeting survivin has received orphan drug certification from FDA, as it can both stimulate T cell immunity and inhibit the activity of survivin pathway. In a phase I clinical trial of 9 survivin-positive patients with rGBM, the patients were treated with SurVaxM, and as a result, 6 of them had a cellular response and 3 of them achieved a local response. The safety of SurVaxM was generally favorable. The median PFS of all patients was 17.6 weeks, and the median OS reached 88.6 weeks with 7 patients surviving more than 12 months [111]. In 2020, the results of the phase II clinical trial of SurVaxM peptide vaccine were published. The study recruited a total of 63 patients diagnosed with primary GBM and the outcome indicated the effectiveness of SurVaxM [111]. For patients with rGBM, another phase II study combined SurVaxM and Pembrolizumab, a humanized monoclonal anti-PD1 antibody that has been comprehensively investigated in various solid tumors, for treatment [112]. All patients were divided into two groups: Arm A included patients who were already resistant to chemotherapy and had not yet received immunotherapy, while Arm B was those who had developed resistance to anti-PD-1 therapy in the previous treatment. The trial has stopped recruiting new testers, and is scheduled to complete in 2024.

### Other peptide vaccines

As one of the landmark events in progression of glioma, Isocitrate dehydrogenase type 1 (IDH1) mutations have been constantly studied as a potential therapeutic target, among which IDH1(R132H) is the most frequent one. Researchers found an immunogenic epitope in IDH1(R132H), indicating it as a favorable target for mutation-specific vaccination to activate anti-tumor immunity, which has led to the initial peptide vaccine [113]. In recent clinical researches, TAA-specific immune responses were found to be superimposedly elicited with more than one peptide in most patients, some of which had significantly prolonged PFS after treatment initiation and demonstrated radiographic tumor responses [85, 107, 113]. The optimized SL-701 vaccine was an interesting attempt, with peptides targeting IL-13Ra2, survivin, and EphA2. The Stemline Therapeutics-established project is currently undergoing a multicenter phase I/II study in adult patients with rGBM [114, 115]. The primary outcomes of the trial are safety, 12-month OS, as well as the objective response rate. Similarly, a multipeptide vaccine named IMA950 has been ongoing a phase I/II trial, which contains 9 major MHC class I and 2 MHC class II peptides. The vaccine, administered with poly-ICLC, was first injected to 19 patients involved in this trial, with 84.6% of them showing tumor-peptide specific CD4 + T-cell responses and a median OS of 19 months. A combination of Pembrolizumab was designed to the follow-up study [116]. Another newly-enrolled phase I/II ROSALIE study conducted on rGBM has released its interim data recently [117]. 76 patients have been included in the clinical trial with the vaccination of EO2401, a polypeptide vaccine originated from three TAAs, IL13Ra2, BIRC5/surviving and FOXM1, partly combined with BEV and nivolumab, a monoclonal anti-PD-1 antibody. All patients have been well tolerated through injections, and the biosecurity feature of EO2401 was almost the same as nivolumab. Robust immune response has been observed in EO2401 plus nivolumab group, while additional standardized BEV was positive for PFS, which indicated an exciting clinical outcome of the combined therapy of EO2401, nivolumab and BEV for follow-up study.

Another important theoretical hypothesis is that once those immune responses involving antigen-positive tumor cells could be generated to multiple peptides, it could be possible to prevent the growth of antigen-negative tumor cells, further amplifying the efficacy of the vaccine. A subsequent phase I study on 14 kinds of HLA-A24–restricted vaccine candidates (ITK-1) has enrolled 12 patients with rGBM based on this approach, demonstrating a prolonged OS of 10.6 months and a similar PFS of 2.3 months, despite the concern of the scale of the study weakening the strength of evidence [118]. Another vaccine of multiple peptides combined TAAs with an additional peptide from the TAA Wilms tumor 1 (WT1) [119]. WT1 is a TAA highly overexpressed in GBM, while DSP-7888, a WT1 peptide vaccin e, has gone through a dose-escalation trial and been undergoing a phase III trial combined with BEV in patients with rGBM [120, 121]. In the context of previous studies, a nonrandomized phase II clinical trial enrolled 21 patients with rGBM, in which WT1 was targeted with an HLA-A*2402-restricted, modified 9-mer peptide in Montanide ISA51, and patients received intra-dermal injection for 12 weeks until progression. The primary outcomes indicated that the vaccine was tolerated and possessed a clinical response, with a 6-month PFS rate of 33.3%, in spite of the unexpected unaltered frequency of WT1 cytotoxic T lymphocytes after immunization [122]. Personalized peptide vaccination (PPV) has become a worthwhile therapeutic choice in several malignances for a more precise and individualized treatment [123]. A randomized phase III trial of PPV on rGBM was completed in 2019, in which 4 of 12 warehouse peptides were selected and assembled into PPV, based on preexisting peptide-specific immunoglobulin G levels. Although the primary and secondary outcome was neither reached, with a median OS of 8.4 months in PPV group versus 8.0 months in placebo group and no statistical difference on median PFS between the two groups, some specific peptides, biomarkers and clinical factors was found to be correlated to a poor survival, such as SART2-93 [96, 124]. Such evidences could be valuable for the design of other PPV and subsequent clinical researches.

### Dendritic cells vaccine

Dendritic cell (DC) is the most important kind of APC in the human immune system. It’s strongly characterized by the ability to stimulate primary T cells to proliferate. After stimulation is applied, DCs could mature and migrate to draining lymph nodes for the induction of immune responses [114]. But the activated T cells won’t divide fast enough to wipe out the cancer cells under nature status. Therefore, researchers tried to generate autologous DCs ex vivo, then treated them with tumor antigens for pre-condition, and finally injected the cells back into patients as an immunotherapy [125]. DCVax-L was developed through this procedure. DCVax-L is an autologous DC vaccine, composed of DCs pulsed with a lysate derived from the patient's own resected tumor, which activates the immune response through a “multiplier effect”. The clinical trial on DCVax-L started to recruit in 2007 and finished in 2015, with 331 newly diagnosed patients from 94 medical centers in 4 countries [95]. Each patient could choose to accept DCVax-L again if a recurrence occurred. The inspiring result of the phase III trial was published this year, that for the 64 rGBM patients using DCVax-L, the median OS was 13.2 months while the external control group was 7.8 months, which was a statistically significant prolongation. The long-term survival was also greatly improved, that the 24 months overall survival was 20.7% versus 9.6% and the 30 months survival was 11.1% versus 5.1%. This has been the first study to succeed in prolonging the OS of rGBM in the past 27 years, which would sure to be one of the landmark clinical trials for GBM and rGBM [126]. Despite some doubts on the analysis methodology and the unclear mechanism of the vaccine, DCVax-L has proved its great potential value for clinical application.

**Fig. 3:**
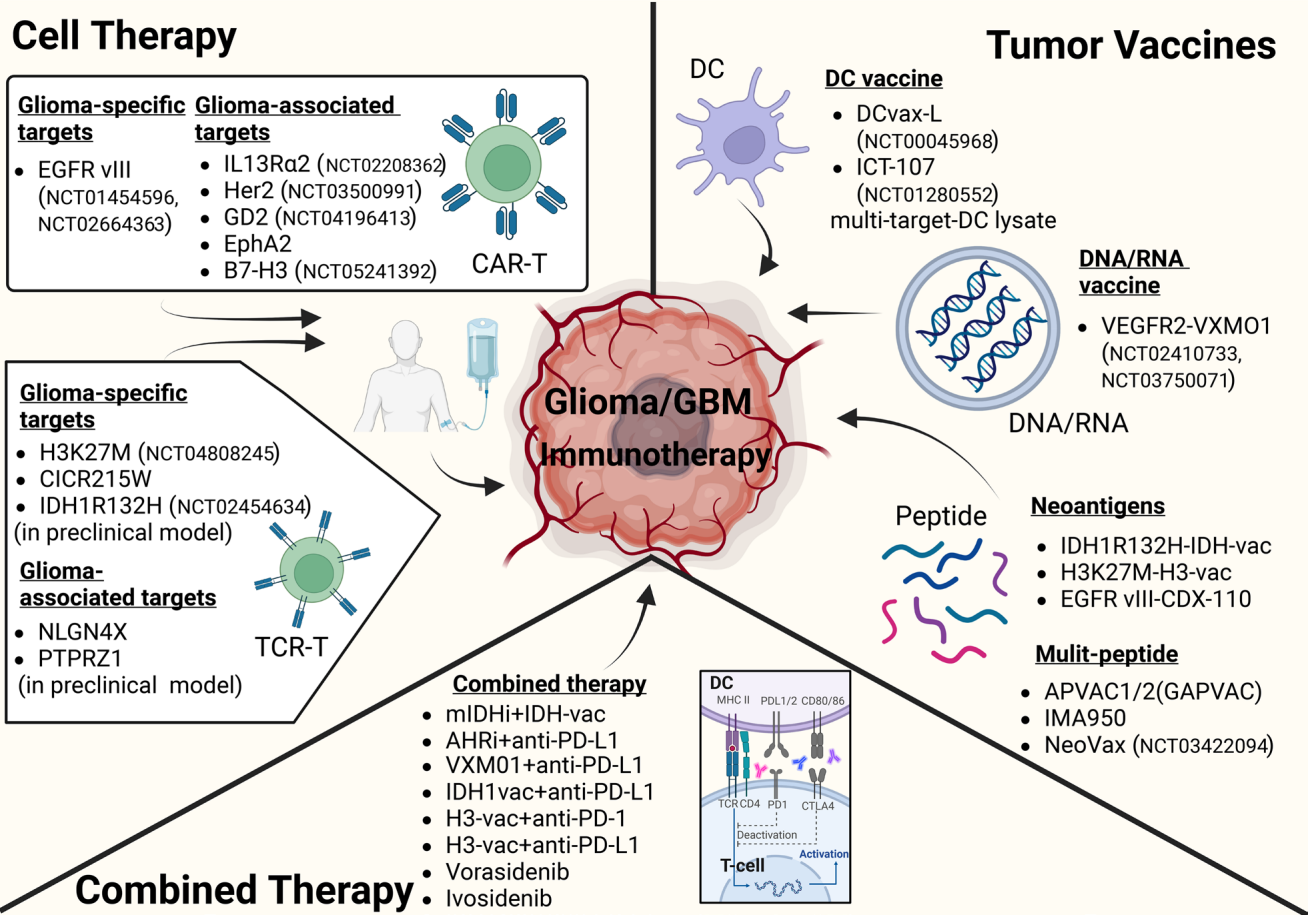
Available modalities and targets of immunotherapy for glioma/glioblastoma. Cell therapy mainly involves CAR-T and TCR-T therapy. As for cell therapy, the glioma-specific and glioma-associated targets for CAR-T therapy includes EGFR VIII (NCT01454596, NCT02664363, NCT02209376, NCT03283631) and IL-13Rα2 (NCT02208362), Her2 (NCT03500991), GD2 (NCT04196413), EphA2, B7-H3 (NCT05241392, NCT04077866), (NCT05241392, NCT05366179); the glioma-specific and glioma-associated targets include H3K27M (NCT04808245), CICR215W, IDH1R132H (NCT02454634) and NLGN4X, PTPRZ1. Tumor vaccines mainly include DC vaccine, DNA/RNA vaccine, neoantigens and peptide targets. As for vaccines immunotherapy, DC vaccine includes DCvax-L (NCT00045968) and ICT-107 (NCT00045968); DNA/RNA vaccine include VEGFR2-VXMO1 (NCT02410733, NCT03750071); neoantigen as peptide targets includes IDH1R132H-IDH-vac (NCT03343197, NCT04056910, NCT02073994, NCT04195555), H3K27M-H3-vac (NCT04943848, NCT04749641, NCT04808245), EGFR VIII-CDX-110 (NCT02573324, NCT01520870, NCT01480479, NCT01498328), multi-peptide targets include APVAC1/2 (GAPVAC) (NCT03422094, NCT02287428), IMA950, NeoVax (NCT03422094). The combined therapy indicates the combination of two or more lines of immunotherapy (including checkpoint inhibitors) as well as small molecule mutant IDH inhibitors. Briefly, there are mIDH inhibitors (mIDHi) + IDH-vac (NCT03750071), AHRi + anti-PD-L1 (NCT03893903), VXM01 + anti-PD-L1, IDH1vac + anti-PD-L1, H3-vac + anti-PD-1 (NCT02960230), H3-vac + anti-PD-L1 (NCT02960230), vorasidenib/ivosidenib/BAY1436032 (NCT02481154, NCT02746081, NCT03030066, NCT03343197, NCT04164901). CAR, chimeric antigen receptor; TCR, T cell receptor-T; DCs, dendritic cells; PD-1, programmed cell death 1; PD-L1, programmed cell death ligand 1

Other DC vaccines, and peptide-pulsed DC vaccines, have been in progress as well [127–130]. ICT-107 is a vaccine of multi-epitope-pulsed autologous DCs with a variety of synthetic TAAs, including AIM-2, MAGE-1, TRP-2, HER-2, and IL-13Rα2. The initial single arm phase I trial contained 17 patients with GBM treated with ICT-107, reaching a median OS of 38.4 months, which was found to be connected to the immune responses to some of the TAAs, further explaining the mechanism behind [131]. In another phase I/II study, a vaccine with αDC1 loaded with synthetic peptides for glioma-associated antigens epitopes and administered with an adjuvant named poly-ICLC was injected to 22 patients with rGBM [132, 133]. 9 of them reached a PFS of more than 12 months, with positive immune responses observed in 58% of the patients, one of which demonstrated a sustained complete response [134]. Jethro et al. tried an autologous DC vaccine pulsed with lysate derived from a GBM stem-like cell line for 35 patients with GBM, including 25 with rGBM [135]. The safety and tolerability were checked and the median OS and PFS were 11.97 months and 3.23 months, both better than the average. A GSC-pulsed DC vaccine (GSC-DCV) was studied in 21 patients with GBM including 10 with rGBM in a phase II trial, conducted by Yu et al. which had a median OS of 10.7 months, as well as a surprising median PFS of 6.9 months [136, 137]. Autologous tumor lysate-pulsed DC vaccine with other specific tumor didn’t show an impressive result. For Gliadel Wafer plus lysate-pulsed DC vaccine at the stage of phase I trial, 17 patients with rGBM reached a median OS of 10.9 months and a median PFS of 1.9 months [138]. Sakai et al. [139] performed a trial for 10 rGBM patients treated with Wilms’ tumor 1-pulsed DC vaccination. After the final injection, however, all the enrolled patients had a progression in tumor. To treat DC vaccine as an adjuvant therapy could be a possible plan as well since DCs may act as immune-boosting adjuvants [94, 140]. Vleeschouwer et al. [141] conducted a phase II trial using adjuvant DC vaccination on 56 adults and children with rGBM. The median OS was about 9.6 months, with a 2-year OS rate of 14.8%. Compared to other cell vaccines, such as whole tumor vaccines [142, 143], DC-based vaccines are showing a good effect on improving the survival of rGBM patients and delaying tumor progression, which could be expected to be one of the most universally pursued vaccination approaches in the future. Major available modalities and targets of the immunotherapy have been summarized in Fig. 3.

### Future challenges

rGBM could be considered as the progressive outcome of patients with GBM. Consequently, it will show a more serious resistance to radiotherapy and chemotherapy and the patients are usually under a worse condition so a lot of them do not have a second chance of operation. Immunotherapy has become an expected new attempt for patients with rGBM, while tumor vaccination is one of the most promising approaches. Despite the disappointing result of the recently released data of clinical trials, especially extensively-concerned phase III trials with immune checkpoint inhibitors PD-1/PD-L1, CTLA-4, CAR-T therapy and viral therapy, tumor vaccine has shown its potential with the new understanding of TAAs and immunosuppressive mechanism of tumor microenvironment.

Multiple phase III trials of tumor vaccine with rGBM are underway, on the basis of solid preclinical studies and clinical trials of early-phase. The safety, tolerance and efficacy have been initially proved in these novel vaccines, however, the low tumor mutational burden and high heterogeneity of rGBM could lead to limited effective therapeutic targets and failure in the following clinical trials, just as those happened in immune checkpoint blockade and CAR-T therapy in the brain tumor. The exciting success of DCVax-L is worthy of reference, but the data analysis method remains questioned, Since the biological mechanism of these immunotherapy have mostly been proved in experiments, a combination of different approaches could be a future direction of the development of tumor vaccines, just as the ongoing clinical trial that combined ATL-DC, a DC vaccine, with checkpoint inhibitor pembrolizumab, hoping to activate a synergistic effect of the systemic antitumor response [144, 145]. Novel adjuvants have been invented and found to boost the effectiveness of the vaccine through engagement of innate immune activation pathways, which is a model for other similar researches. The design of clinical trial can also be a solution, by setting a more divided subgroup for research, choosing different clinical outcomes from a wider range and cooperation between clinical institutions. It is certain that more tumor vaccines will reach the stage to test their efficacy, and a more complicated pathway can be applied in newly-designed vaccines, as well as more exquisite therapeutic strategies based on individualized vaccination, making it easier to overcome the present immunosuppressive microenvironment and antitumor immunity of rGBM.

## Conclusion

GBM is the most malignant CNS tumor with a high recurrence rate. Multiple tumor vaccines have been proved safe and tolerated in patients with rGBM through preclinical and early-phase trials, with several large-scale phase III studies ongoing. Despite the past failure of other immunotherapies in GBM, tumor vaccination is considered to be promising due to the recent progress in studying the immunosuppressive mechanism of GBM. The immunological feature of rGBM has not been fully understood, which shows some common features of GBM and brain tumors, as the intrinsic resistance, adaptive resistance and other type of resistance among all phases of the antitumor immune response. rGBM itself also demonstrates some unique characteristics, as it prefers the PN subtype and seems to have an increasing immune infiltration, which could partly explain the poor prognosis of rGBM, while it also indicated the possibility of using tumor vaccination to create more effective therapeutic therapies. More details on the microenvironment remain to be explored, and more newly-designed tumor vaccines will promote the development of better therapy for patients with rGBM.

## Data Availability

All data and materials used are available from the corresponding author upon reasonable request.
